# Abnormal glucose regulation in patients with acute ST- elevation myocardial infarction-a cohort study on 224 patients

**DOI:** 10.1186/1475-2840-8-6

**Published:** 2009-01-30

**Authors:** Eva C Knudsen, Ingebjørg Seljeflot, Michael Abdelnoor, Jan Eritsland, Arild Mangschau, Harald Arnesen, Geir Ø Andersen

**Affiliations:** 1 Center for Clinical Heart Research, Ullevål University Hospital, University of Oslo, Oslo, Norway; 2 Department of Cardiology, Ullevål University Hospital, University of Oslo, Oslo, Norway; 3 Center of Clinical Research, Unit of Epidemiology and Biostatistics, Ullevål University Hospital, University of Oslo, Oslo, Norway

## Abstract

**Background:**

A high prevalence of impaired glucose tolerance and unknown type 2-diabetes in patients with coronary heart disease and no previous diagnosis of diabetes have been reported. The aims of the present study were to investigate the prevalence of abnormal glucose regulation (AGR) 3 months after an acute ST-elevation myocardial infarction (STEMI) in patients without known glucometabolic disturbance, to evaluate the reliability of a 75-g oral glucose tolerance test (OGTT) performed very early after an acute STEMI to predict the presence of AGR at 3 months, and to study other potential predictors measured in-hospital for AGR at 3 months.

**Methods:**

This was an observational cohort study prospectively enrolling 224 STEMI patients treated with primary PCI. An OGTT was performed very early after an acute STEMI and was repeated in 200 patients after 3 months. We summarised the exact agreement observed, and assessed the observed reproducibility of the OGTTs performed in-hospital and at follow up. The patients were classified into glucometabolic categories defined according to the World Health Organisation criteria. AGR was defined as the sum of impaired fasting glucose, impaired glucose tolerance and type 2-diabetes.

**Results:**

The prevalence of AGR at three months was 24.9% (95% CI 19.1, 31.4%), reduced from 46.9% (95% CI 40.2, 53.6) when measured in-hospital. Only, 108 of 201 (54%) patients remained in the same glucometabolic category after a repeated OGTT. High levels of HbA1c and admission plasma glucose in-hospital significantly predicted AGR at 3 months (p < 0.001, p = 0.040, respectively), and fasting plasma glucose was predictive when patients with large myocardial infarction were excluded (p < 0.001).

**Conclusion:**

The prevalence of AGR in STEMI patients was lower than expected. HbA1c, admission plasma glucose and fasting plasma glucose measured in-hospital seem to be useful as early markers of longstanding glucometabolic disturbance. An OGTT performed very early after a STEMI did not provide reliable information on long-term glucometabolic state and should probably not be recommended.

## Background

Several prospective studies have reported a high prevalence of impaired glucose tolerance (IGT) and unknown type 2-diabetes (DM) in patients with coronary heart disease and no previous diagnosis of DM [1-4] although data on patients with acute ST-elevation myocardial infarction are scarce. IGT and DM have been shown to be strong risk factors for future cardiovascular events after acute myocardial infarction [5]. Recent European guidelines on DM, prediabetes, and cardiovascular disease recommend that patients without known diabetes, but with established cardiovascular disease should be investigated with an oral glucose tolerance test (OGTT) [6]. However a scientific statement from the American Heart Association Diabetes Committee of the Council on Nutrition, Physical Activity, and Metabolism do not encourage routine use of such screening [7]. In the European guidelines there is no consensus about the timing of an OGTT performed after an acute myocardial infarction [6]. Early testing may be confounded by stress reactions accompanying an acute myocardial infarction, but could be important in order to identify patients with cardiovascular events in the first weeks after an acute myocardial infarction [8]. The Glucose tolerance in Acute Myocardial Infarction (GAMI) study indicated that an OGTT performed early (day 4–5) after an acute myocardial infarction [1] provided reliable information on long-term glucometabolic state [9].

Routine use of OGTT, as stated by the European guidelines, in the follow-up of patients with myocardial infarction has so far not been implemented in Norway. Rapid transfer of stable patients after primary PCI from tertiary centers to community hospitals makes it difficult to implement the new guidelines into daily-life practice. This study was undertaken to investigate the prevalence of unknown DM, IGT and impaired fasting glucose (IFG) in patients with STEMI during stable conditions after 3 months of follow-up and to evaluate the reliability of performing an OGTT-based glucometabolic classification in-hospital in order to predict glucometabolic disturbance defined by a repeated OGTT at 3 months. The study had a practical approach: Performing a very early OGTT at the PCI center in stable patients, before transfer (usually within 24 hours) back to community hospitals, in order to establish screening for unknown DM and IGT in STEMI patients in our community. Finally, in an explanatory strategy, to study various biomarkers and risk factors identified during the initial hospitalisation as possible predictors of DM and IGT defined at 3 months.

## Methods

### Study population

The study was designed as an observational cohort study prospectively including STEMI patients treated with primary PCI admitted to the coronary care unit at Ullevål University Hospital, Oslo, Norway, between November 2005 and May 2007. The OGTT was performed after overnight fasting, before transfer from the coronary care unit back to the referring hospitals. At Ullevål University Hospital, stable patients are regularly returned within 24-hours after treatment. At enrolment, patients were clinically stable, without persistent glyseroltrinitrat infusion, chest pain, nausea or symptoms of heart failure. Patients with known DM, serum creatinine concentration ≥ 200 umol/L, and age > 85 years were excluded. The OGTT was not performed in patients with persistent hyperglycaemia in order to avoid further glucose overloading.

The Regional ethics committee approved the study and all patients provided written informed consent. STEMI was defined as ST-segment elevation of > 2 mm in two or more contiguous chest leads *or *> 1 mm in two or more limb leads *or *new left bundle-branch block, together with typical symptoms (chest pain or discomfort > 20 min).

### Laboratory methods

Admission plasma glucose concentration was analysed from a blood sample taken in the catheterisation laboratory as soon as possible after PCI. After overnight fasting, blood samples for routine analysis by use of conventional methods including plasma glucose, were drawn. A standardised OGTT (2 h) with 75 g of glucose dissolved in 200 mL water was performed [10]. The median time from balloon to OGTT was 16 hr and 35 min. The OGTT was repeated after three months.

Serum-cTroponin T was measured by electrochemiluminescence technology for quantitative measurement (3^rd ^generation cTroponinT, Elecsys 2010, Roche, Mannheim, Germany). The lower detection limit of the assay is 0.01 ug/l with a recommended diagnostic threshold of 0.03 ug/l. The inter-assay coefficient of variation was 7%.

Urinary albumin excretion was assessed in a morning spot urine sample and expressed as albumin/creatinine ratio. Microalbuminuria was defined as 30–300 mg albumin secreted per 24 hours [11].

### Clinical follow-up

At 3 months, the patients underwent an OGTT, clinical examination, and answered a questionnaire about lifestyle changes after discharge from hospital, including weight, smoking habits, and physical activity. Physical activity was reported as 30 min of activity, 0–1 days/week, 2–3 days/week, or > 3 days/week.

### Single Photon Emission Computed Tomography imaging

Left ventricular ejection fraction, end-diastolic and end-systolic volume and infarct size expressed as percent of left ventricular mass, were assessed at rest after 3 months by Single Photon Emission Computed Tomography imaging with technetium 99 m-tetrofosmin [12].

### Classification of glucometabolic state

The different glucometabolic categories were defined according to the World Organisation criteria [13]. Classification of glucometabolic state was primarily based on the result of an OGTT and the patients were divided into one of the following four categories, (glucose levels given in mmol/l):

Normal Glucose Regulation (NGR) = OGTT (0 min) < 6.1 and OGTT (2 h) < 7.8

Impaired Fasting Glucose (IFG) = OGTT (0 min) ≥ 6.1 < 7.0 and OGTT (2 h) < 7.8

Impaired Glucose Tolerance (IGT) = OGTT (0 min) < 7.0 and OGTT (2 h) ≥ 7.8 < 11.1

Type 2-diabetes (DM) = OGTT (0 min) ≥ 7.0 and/or OGTT (2 h) ≥ 11.1

Because of limited number in each category, the patients were also divided into:

Abnormal Glucose Regulation (AGR) = IFG+IGT+DM or NGR.

The patients were also categorised based on the results of fasting plasma glucose (FPG) only:

Normal Glucose Regulation (NGR) = FPG < 6.1

Impaired Fasting Glucose (IFG) = FPG ≥ 6.1 < 7.0

Type 2-diabetes (DM) = FPG ≥ 7.0

### Statistics

In the prevalence study an apriori power analysis was performed. With a hypothesised prevalence of AGR of 60% [1-3] with a precision of 5% and a chosen probability of 90% a sample size of 222 patients was needed to determine the prevalence of unknown AGR. Furthermore, a post hoc power analysis was done for potential in-hospital predictors for the outcome AGR at 3 months showing that 116 patients were required considering a type 1-error of 5%, a power of 80%, a prevalence of AGR in patients with low HbA1c of 17%, and an OR (crude) = 4.15 for the association HbA1c on AGR. Similar analyses were also performed with the associations of fasting plasma glucose and admission plasma glucose on AGR.

Values are presented as median (25, 75% quartiles) or proportions. Mann-Whitney's test was used for comparing groups. In order to identify possible in-hospital predictors of AGR defined at 3 months, we hypothesised an association between the 3 exposition variables HbA1c, fasting plasma glucose, and admission plasma glucose and the outcome AGR. The other variables were potential confounders or effect modifiers of this association. The odds ratio (OR) and its 95% confidence interval (CI) were used to quantify this association. We performed a stratification analysis and dichotomised continuous exposition variables into high and low values using the cut-off point 6.1 mmol/l for fasting plasma glucose according to the literature and the 75% percentile in the present material for the other variables. The Mantel-Haenszel method was used to highlight potential effect modification by the Breslow-Day test of heterogeneity and to quantify potential confounders [14]. Additional information is available online (see additional file 1).

A logistic regression model, including a backward elimination procedure and finally taking in consideration the concept of validity and precisions, performed adjustment for multi-confounders. The STROBE guidelines were followed [15].

Analyses were performed using Epi-info software, 2005, version 3.3.2.

A value of p < 0.05 was considered statistically significant.

## Results

### Baseline and follow up characteristics

Two hundred and twenty five patients without previously known DM or IGT were enrolled in the study. The OGTT was well tolerated, 224 completed an in-hospital OGTT while one patient interrupted the test because of acute nausea. None of the patients complained of chest-pain or underwent a new coronary angiography. No in-hospital deaths occurred. The median time from onset of chest pain to balloon (PCI) was 3 hr and 39 min and 16 hr and 35 min from balloon to OGTT. The OGTT was repeated in 200 patients after 3 months. The reasons for not repeating the test were: death (n = 1), fasting plasma glucose > 7.0 mmol/L (n = 1) and unwillingness (n = 22). Table 1 summarises the baseline characteristics of the study population. Notably, the majority of the patients presented with their first myocardial infarction, 62% had single vessel disease and 92% of the patients were in Killip class 1 (data not shown). Single photon emission computed tomography imaging at 3 months showed that 85% of the patients had an left ventricular ejection fraction > 50%.

**Table 1:** Baseline characteristics of the total population (n = 224)

	Patients
Age (years)	58 (51, 67)
Male	185 (82.6%)
Previous disorder:	
Myocardial infarction	16 (7.1%)
Angina pectoris	7 (3.1%)
Hypertension (treated)	58 (25.9%)
Hyperlipidaemia (treated)	20 (8.9%)
Status at baseline	
Current smoker	109 (48.7%)
BMI (kg/m2)	26 (24.4, 28.7)
Waist circumference (cm)	100 (94, 107)
Stent in culprit lesion	215 (96.0%)
Gp IIb/IIIa antagonist treated	79 (35.3%)
Single -coronary vessel disease	139 (62.1%)
Double-coronary vessel disease	64 (28.6%)
Triple-coronary vessel disease	21 (9.4%)
Time from symptoms to balloon (min)	219 (140, 378)
Time from PCI to OGTT (min)	995 (689, 1277)
Medication at discharge from CCU	
Aspirin	224 (100%)
Clopidogrel	222 (99.1%)
β-blockers	181 (80.8%)
Lipid lowering agents	221 (98.7%)
Angiotensin converting enzyme-inhibitors	36 (16.1%)
Angiotensin II-receptor blockers	18 (8.0%)
LVEF^a^	64 (56, 70)

Data are presented as median with inter-quartile range or proportions.

BMI: body mass index, LVEF: left ventricular ejection fraction. ^a^LVEF diagnosed 3 months after discharge.

At 3 months 25% of the patients had stopped smoking, 41% had increased the number of days per week of physical activity, 36% had lost weight and 30% had gained weight. The proportion of AGR in patients who had lost weight and in patients who had gained weight was 28% and 25%, respectively (data not shown).

### Prevalence of AGR

**Figure 1:**
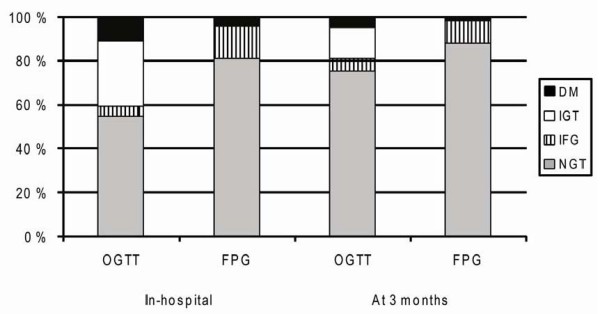
**Glucometabolic classification of 201 STEMI patients based on the results of an OGTT or fasting plasma glucose only, in-hospital and at 3 months**. FPG: fasting plasma glucose, IFG: impaired fasting glucose, IGT: impaired glucose tolerance, NGR: normal glucose regulation, OGTT: oral glucose tolerance test, DM: type 2-diabetes.

The median plasma glucose concentration at admission was 6.9 mmol/L ((25, 75% percentiles) 6.0, 7.8), the median fasting plasma glucose was 5.3 mmol/L (4.9, 5.9) and the median HbA1c value was 5.5% (5.3, 5.8) (n = 207). Figure 1 shows the prevalence of NGR, IFG, IGT and DM in-hospital and at 3 months, based on a glucometabolic classification by OGTT and alternatively, by fasting plasma glucose alone. Based on the given categories we defined, abnormal glucose regulation (AGR) as the sum of IFG, IGT and DM, in spite of different risk profiles between IFG, IGT and DM. The prevalence of AGR after an OGTT- based classification was 46.9% (95% CI 40.4, 53.9) (n = 224) in-hospital and 24.9% (19.1, 31.4) (n = 201) at 3 months, respectively.

Table 2 compares the results of an OGTT- based glucometabolic classification of the patients in-hospital and at 3 months. Fifty-four % of the patients remained in the same glucometabolic category after a repeated OGTT. Only 5 out of 22 patients defined as diabetic in-hospital remained in the diabetic category after 3 months.

**Table 2:** Glucometabolic classification by OGTT in patients with ST-elevation infarction in-hospital (OGTT1, row), and at 3 months (OGTT2, column)

	NGR (OGTT2)	IFG (OGTT2)	IGT (OGTT2)	DM (OGTT2)	Total (OGTT1)
NGR (OGTT1)	91	6	11	1	109

IFG (OGTT1)	8	1	1	0	10

IGT (OGTT1)	41	4	11	4	60

DM (OGTT1)	11	0	6	5	22

Total (OGTT2)	151	11	29	10	201

Data are number of patients.

Observed concordance: 53.7%

One patient was categorized based on the results of fasting plasma glucose only, because of persistent hyperglycaemia.

### Predictors of AGR

In Table 3, patient characteristics assessed at hospitalisation are compared between the NGR group and the AGR group, as defined by an OGTT three months later. Patients with AGR were older, there were significantly more women, and they had significantly higher levels of HbA1c, admission plasma glucose, and fasting plasma glucose.

**Table 3:** Clinical and laboratory characteristics of patients in-hospital according to glucometabolic category defined by OGTT at 3 months

	NGR (n = 151)	AGR (n = 50)	P
Age (years)	57 (51, 65)	61 (52, 72)	0.037
Male	129 (85.4%)	36 (72.0%)	0.032
Current smoker	67 (44.4%)	24 (48.0%)	0.656
Treated hypertension	38 (25.2%)	17 (34.0%)	0.226
BMI (kg/m^2^)	26.2 (24.3, 28.7)	26.1 (24.6, 28.7)	0.741
Cholesterol (mmol/l)	5.1 (4.4, 5.7)	5.1 (4.4, 5.7)	0.839
Triglycerides (mmol/l)	1.27(0.89, 1.79)	1.25 (0.96, 1.83)	0.623
HDL-cholesterol (mmol/l)	1.19 (0.97, 1.43)	1.12 (1.00, 1.35)	0.659
Serum uric acid (mmol/l)	334 (289, 382)	347 (282, 409)	0.288
Microalbuminuria	26 (18.2%)	10 (22.2%)	0.549
scTnT (ug/l)	4.85 (2.45, 8.31)	4.97 (2.38, 10.25)	0.469
HbA1c (%)	5.5 (5.3, 5.7)	5.8 (5.5, 6.0)	< 0.001
Admission plasma glucose (mmol/l)	6.6 (5.8, 7.5)	7.4 (6.6, 8.7)	<0.001
FPG (mmol/l)	5.2 (4.9, 5.7)	5.6 (5.1, 6.3)	0.003

Data are presented as median with inter-quartile range or proportions.

AGR: abnormal glucose regulation, BMI: body-mass index, FPG: fasting plasma glucose, NGR: normal glucose regulation, scTnT: serum-cTroponinT.

In univariate analyses HbA1c, admission plasma glucose and fasting plasma glucose significantly predicted the presence of AGR at 3 months (p < 0.001, p = 0.006 and p = 0.011, respectively) (Table 4). HbA1c (p < 0.001) and admission plasma glucose (p = 0.040), analysed separately, remained significantly predictive after adjustment for confounders (Table 4). When analysing on fasting plasma glucose concentration as an independent biomarker, serum-cTroponinT modified the effect of fasting plasma glucose on AGR, thus the results were stratified into two subgroups, and as shown in Table 4, fasting plasma glucose was independently predicting AGR at 3 months only in patients with serum-cTroponin T below the highest quartile (< 8.80 ug/l), with an adjusted OR 5.50 (95% CI 2.00, 15.10).

**Table 4:** Logistic regression analysis of HbA1c, admission plasma glucose and fasting plasma glucose as independent predictors in-hospital for AGR defined by an OGTT at 3 months

	AGR		AGR		AGR scTnT > 8.80 ug/l (n = 51)		AGR scTnT < 8.80 ug/l (n = 150)	
	OR (crude) (95% CI)	P	OR (adjusted) (95% CI)	P	OR (adjusted) (95% CI)	P	OR (adjusted) (95% CI)	P

HbA1c^a^ > 5.7%	4.15 (2.03, 8.48)	<0.001	3.76 (1.82, 7.79)	<0.001				
Admission plasma glucose^b^ > 7.7 mmol/l	2.59 (1.31, 5.12)	0.006	2.12 (1.03, 4.33)	0.040				
FPG ^c^ ≥ 6.1 mmol/L	2.62 (1.25, 5.50)	0.011			0.69 (0.15, 3.14)	0.636	5.50 (2.00, 15.10)	<0.001

AGR: abnormal glucose regulation, FPG: fasting plasma glucose, scTnT: serum-cTroponinT.

^a^Adjusted for gender and age.

^b^Adjusted for age.

^c^scTnT > 8.80 ug/l (highest quartile) was an effect modifier on the association FPG on AGR, thus data are presented in 2 subgroups, both adjusted for age, uric acid and gender.

## Discussion

### Prevalence of AGR

The main findings in the present study were that only 25% of the patients were classified by OGTT as having AGR in a clinically steady situation 3 months after an acute STEMI and only about 5% had previously unknown DM. About 50% of the patients with STEMI were classified as having undetected AGR based on an OGTT classification in-hospital, but only half of these patients remained in the AGR group after a repeated OGTT 3 months later. Using fasting plasma glucose classification only, about 50% of the patients with AGR would have been misclassified, both in-hospital and after 3 months. To disclose the actual glucometabolic state in these patients it seems necessary to perform an OGTT. The latter result is in line with a previous study, showing that two-thirds of the patients with undiagnosed diabetes would have been missed using fasting plasma glucose classification alone [2].

In the present study the OGTT was performed very early after a PCI treated STEMI and an initially higher prevalence of AGR could be expected as an acute STEMI is accompanied by a substantial release of stress hormones. However, we found a somewhat lower prevalence of AGR than reported in previous studies, both in-hospital and at 3 months. Three prospective studies on mixed populations of patients with acute and stable coronary artery disease have shown that about 60% of the patients had undiagnosed AGR [1-3]. A repeated OGTT was performed only in one of these studies showing that 66% of the patients had AGR before hospital discharge [1] and the prevalence was reported to be similar after 3 and 12 months [9]. In the present study the prevalence of AGR at 3 months was much lower, which the selected population of STEMI patients may partly explain. The patients were mainly Caucasians, relatively young, included a low number of women, a high proportion of first acute myocardial infarction, and a preponderance of single vessel disease. Additionally, patients with persistent hyperglycaemia were excluded. It is possible that the prevalence of AGR after 3 months would have been higher if these patients had been included in the 3 months follow-up.

The prevalence of AGR was reduced by about 50% after 3 months indicating poor reliability of the early classification. The measured in-hospital prevalence of AGR reflects not only long-standing glucometabolic disturbance, but also an acute stress epiphenomenon as discussed.

### OGTT in patients with STEMI

Patients with acute myocardial infarction and diabetes have a higher short and long-term mortality rate than non-diabetic patients [16]. The 2-hour blood glucose has been shown to be superior to fasting blood glucose to predict the risk of future cardiovascular disease and death from all causes in individuals with hyperglycaemia [17]. The present study revealed that a very early OGTT in STEMI-patients was well tolerated, except in one patient. None of the patients died in-hospital, reflecting the intention to exclude unstable patients.

To examine the reproducibility of an OGTT, two independent tests should be performed within a short period of time in stable patients without persistent acute illness[10]. Even under these circumstances previous studies have suggested poor reproducibility of an OGTT [18]. In the present study we compared the OGTT performed after initial stabilisation of acute illness, and stable disease, thus we did not test the reproducibility of the OGTT, but evaluated the reliability of an early glucometabolic classification by OGTT by repeating the test after 3 months. Generally, the variation in the repeated OGTT results has to a great extent been explained by the random variation of plasma glucose concentration [19]. In our study the variation in the OGTT results after 3 months of follow up to a great extent may have been influenced by the different test situation mentioned. This is in line with another study showing that acute myocardial infarction induces hyperglycaemia and give rice to insulin resistance [20]. These investigators demonstrated that blood glucose levels and HOMA-IR decreased significantly during hospital stay with no further decrease between discharge (day 4–5) and 3 months of follow up reflecting that acute illness influence the very early rise and fall in glucose levels in-hospital [20].

Only 54% of the patients in our study were classified into the same glucometabolic state on both occasions which is in accordance with results from other studies performing repeated OGTTs [20,21]. The intra-individual tracking of oral glucose tolerance in our study was poor, but comparable to what was found in the GAMI study (49 and 54%, respectively) [20]. In the present study, 11 out of 22 patients classified as having diabetes in-hospital converted to NGR at 3 months. These results underline the World Health Organisation recommendation for a repeated OGTT under stable conditions to confirm or exclude an abnormality of glucose regulation in asymptomatic individuals [10]. Nevertheless, individuals with one abnormal OGTT have been shown to have a higher cardiovascular risk profile compared to individuals who had two normal OGTTs [18] and a single positive OGTT in patients with acute myocardial infarction seems to identify patients at higher risk of future cardiovascular event [5]. The present study do not address whether an early OGTT after a myocardial infarction can identify patients with worse prognosis.

### Predictors of AGR

In order to identify patients with AGR after 3 months without performing an in-hospital OGTT, analysis of risk markers was undertaken. We could not demonstrate any differences in baseline values of conventional cardiovascular disease risk factors between the AGR and NGR groups, classified after 3 months. However, a high HbA1c value and a high admission plasma glucose value in-hospital were independently predicting AGR at 3 months with an adjusted OR of 3.76 and 2.12, respectively. Furthermore, a high fasting plasma glucose value in-hospital, strongly predicted AGR at 3 months (adjusted OR 5.50), when patients with large myocardial infarction were excluded indicating that an acute stress reaction may dominate in patients with a large acute myocardial infarction.

This is in accordance with other studies showing HbA1c values at admission and fasting plasma glucose at discharge from hospital (day 4 after acute myocardial infarction) to be independent predictors of AGR at 3 months [1].

### Clinical implication

The European guidelines recommend that patients without known diabetes, but with established cardiovascular disease should be investigated with an OGTT [6]. In order to try to implement the new guidelines in our region we performed an OGTT before transfer to referring hospitals. The present results demonstrate that it is safe to perform a very early OGTT in stable patients with acute STEMI, but a very early classification did not provide reliable information on long-term glucometabolic state, and the present results do not encourage routine use of an OGTT at this early time point in acute STEMI patients However, a substantial proportion of the patients had undetected AGR after 3 months and it is important to diagnose these patients and offer them a close follow- up on lifestyle interventions and optimal medical treatment in order to reduce events in this high-risk population.

### Limitations

Unstable patients and patients with persistent hyperglycaemia in-hospital were excluded from the study to avoid further glucose loading, possibly making a selection bias towards more glucometabolically normal patients. This may have contributed to the somewhat unexpected low prevalence of undetected AGR in STEMI patients.

## Conclusion

The prevalence of AGR in a STEMI-population was lower than previously reported in patients with acute myocardial infarction. The prevalence was relatively high when measured in-hospital by a very early OGTT, but half of these patients were categorised as having normal glucose regulation when re-tested at 3 months. Based on these results an OGTT should probably not be recommended very early after an acute STEMI whereas an OGTT should be performed during follow-up in order to identify patients with persistent glucometabolic disturbances. High levels of HbA1c, admission plasma glucose and fasting plasma glucose (except in patients with large myocardial infarction) measured in-hospital predicted an increased risk of AGR defined at 3 months, indicating that these biomarkers can be used as early markers of a long standing glucometabolic disturbance in patients with acute myocardial infarction.

## Competing interests

The authors declare that they have no competing interests.

## Authors' contributions

ECK performed the statistical analysis of the data presented and drafted the manuscript. MA made substantial contribution with statistical analysis. GØA contributed with the conception and design of the study. ECK, IS, MA, JE, AM, HA and GØA participated in the study design and interpretation and revised the manuscript critically for important intellectual content. All authors have read and approved the final manuscript.

## Supplementary Material

Additional file 1 **Table A.** Stratified analysis on the association between the 3 exposition variables HbA1c, fasting plasma glucose, and admission plasma glucose and the outcome abnormal glucose regulation on major potential confounders using the Mantel-Haenzel method. Click here for file
