# Supplementary material for: Abnormal glucose regulation in patients with acute ST- elevation myocardial infarction-a cohort study on 224 patients
Source: Cardiovasc Diabetol. 2009 Jan 30;8:6. doi: 10.1186/1475-2840-8-6 (PMC2646717; doi:10.1186/1475-2840-8-6)
Supplement: Additional file 1 — Table A. Stratified analysis on the association between the 3 exposition variables HbA1c, fasting plasma glucose, and admission plasma glucose and the outcome abnormal glucose regulation on major potential confounders using the Mantel-Haenzel method. [file 1475-2840-8-6-S1.doc]

## Table A. Stratified analysis on the association between the 3 exposition variables HbA1c, fasting plasma glucose, and admission plasma glucose and the outcome abnormal glucose regulation on major potential confounders using the Mantel-Haenzel method.

| AGR- AGR+ OR ORMH Breslow &Day  95%CI 95%CI Heterogeneity test |
| --- |
| HbA1c HbA1c p-value  (< 5.8 % ≤)  no yes no yes  Age  67 year 93 19 15 13 4.24 (1.74-10.35) 3.72 (1.80-7.70) 0.6476  >67 19 8 8 10 2.97 (0.86-10.30)  Gender  Male 98 21 17 17 4.67 (2.05-10.61) 3.91 (1.90-8.02) 0.4225  Female 14 6 6 6 2.33 (0.53-10.27)  Crude effect 112 27 23 23 4.15 (2.03-8.48) |
| Fasting plasma glucose.  (< 6.1 mmol/L ≤)  no yes no yes  Age  67 year 104 16 18 12 4.33 (1.76-10.66) 2.48 (1.18-5.20) 0.0541  >67 24 7 16 4 0.86 (0.22-3.41)  Gender  Male 111 18 25 11 2.71 (1.14-6.45) 2.45 (1.16-5.20) 0.6789  Female 17 5 9 5 1.89 (0.43-8.30)  scTnT  <8.80 ug/l 105 12 22 11 4.38 (1.71, 11.18) * 0.0448  >8.80 23 11 12 5 0.87 (0.25, 3.09)  High uric acid  <387 umol/l 97 20 21 12 2.77 (1.18-6.53) 2.86 (1.34-6.10) 0.8838  >387 31 3 13 4 3.18 (0.62-16.24)  Crude effect 128 23 34 16 2.62 (1.25-5.50) |
| Admission plasma glucose.  (< 7.8 mmol/L ≤)  no yes no yes  Gender  Male 105 24 21 15 3.13 (1.41-6.94) 2.32 (1.17-4.60) 0.1872  Female 13 9 8 6 1.08 (0.28-4.21)  Age  67 year 101 19 20 10 2.66 (1.08-6.56) 2.08 (1.02-4.23) 0.4298  >67 17 14 9 11 1.48 (0.48-4.59)  Current smoker  No 61 23 13 13 2.65 (1.07-6.56) 2.73 (1.36-5.47) 0.9204  Yes 57 10 16 8 2.85 (0.97-8.41)  High LDL-cholesterol  <4.12 mmol/l 90 20 25 15 2.70 (1.21-6.03)  >4.12 28 13 4 6 3.23( 0.78-13.45) 2.82 (1.40-5.68) 0.8298  Crude effect 118 33 29 21 2.59 (1.31-5.12) |

*Cannot be calculated because serum-cTroponinT (scTnT) is an effect modifier of fasting plasma glucose on abnormal glucose regulation (AGR).

The confounding effect is quantified using the formula ORMH-ORcrude/ ORMH
